# AKT and its related molecular feature in aged mice skin

**DOI:** 10.1371/journal.pone.0178969

**Published:** 2017-06-07

**Authors:** Haiyan Chen, Xusheng Wang, Jimin Han, Zhimeng Fan, Sobia Sadia, Rongrong Zhang, Yingsheng Guo, Yuyang Jiang, Yaojiong Wu

**Affiliations:** 1 Tsinghua Berkeley Shenzhen Institute, Tsinghua University, Shenzhen, China; 2 The Shenzhen Key Laboratory of Health Sciences and Technology, Graduate School at Shenzhen, Tsinghua University, Shenzhen, China; 3 Medical Key Laboratory of Health Toxicology of Shenzhen, Shenzhen Center for Disease Control and Prevention, Shenzhen, China; 4 School of Life Sciences, Tsinghua University, Beijing, China; 5 Department of Nephrology, Shenzhen People's Hospital, Second Clinical Medical College, Jinan University, Shenzhen, China; 6 The Second People’s Hospital of Futian District, Shenzhen, China; NYU Langone Medical Center, UNITED STATES

## Abstract

Previous studies suggest that Akt signaling promotes tissue regeneration and decreased Akt activities are found in aged tissues. However, this study finds that the expression and activation levels of Akt in the mice skin increased with age. Additionally, the expression levels of Pten, p16, p21 and p53 also elevated with increased age. Immuno-fluorescence analysis showed that Akt phosphorylation found in the epidermal cells (with increased levels of NF-κB activation) were also found. In vivo inhibition of AKT activity result in reduced NF-κB activation. Our results suggest that increasing Akt/ NF-κB is a crucial mediator of skin aging, which can increase the susceptibility of cell transformation.

## Introduction

The signaling pathway of insulin/insulin-like growth factor-1 (IGF-1)/phosphatidylinositol-3 kinase (PI3K)/Akt (also known as protein kinase B) has been involved in regulating the longevity of organisms. Mutations that decrease the activity of the insulin/IGF-1 pathway increase the longevity of nematode C. elegans, Drosophila and mice[1–4]. In mammalian cells,activation of Akt has been shown to induce proliferation and survival, while over activated Akt signaling tend to induce cell transformation[5–7]. Previous studies have suggested that Akt signaling promotes tissue regeneration. A decline in long-term regeneration capacity of hematopoietic stem cells has been shown in mice with deficiency of Akt[8]. In addition, Akt activation levels were found to decrease with age and correlate with pancreatic regeneration in mice [9, 10].

The significance of PI3K/Akt in regulating skin stem cells has been found in previous studies. Loss of *Akt1* was found to cause thinner epidermal layer and retarded hair follicle development [11]. Forced Akt phosphorylation in keratin (K) 5 cells in myrAkt-GFP mice was suggested to cause bulge stem cells to exit from quiescence [12]. Similarly, induced Akt activation in the skin caused the activation of hair follicle leading to epidermal hyperplasia in mice [13, 14].

In this study, we found increased expression and activation of Akt in the aging mice skin. In addition, the expression of Pten, p16, p21 and p53 were also elevated. Immuno-fluorescence analysis revealed elevated Akt phosphorylation (p-Akt) in cells in the epidermis and hair follicles, where increased levels of NF-κB activation were found in vivo assay validate the crucial role of Akt/NF-κB signaling of epidermal cell aging. Our results suggest that Akt activation increases in the skin with aging and inflammation thus potentially increase the susceptibility of cell transformation.

## Materials and methods

### Mice

C57/B6 mice (7 weeks old, female and male) were purchased from Guangdong Medical Laboratory Animal Center, Guangzhou, China(http://gdmlac.com.cn). Animals were maintained in a temperature controlled environment (20 ± 1°C). All procedures were approved by the Animal Ethics Committee of Shenzhen Center for Disease Control and Prevention (CDC). C57BL/6 (80days, 6months,18months, 24months) mice were anesthetized with an intraperitoneal injection of sodium pentobarbital (50 mg/kg),all the samples were picked from the back of mice(with hair removal) with a 6mm in diameter skin biopsy punch[15]. When experiments were finished, all the mice were sacrificed by using CO_2._

### Inhibitors

A total of 50mg of perifosine (APExBIO,A8309) incorporated in a 50-g lipid emulsion or an equal amount of lipid emulsion alone (control) was smeared onto the dorsal skin of 12 months old mice after hair depilation at a dose of 1 g per mouse per day for 15 consecutive days. After hair removal, 5 ug bpV (phen), a PTEN inhibitor (BioVision, USA), incorporated into 20 ul matrigel ((BD Biosciences, USA)) was injected intracutaneously into the backs of 80 days mice. Equally, the littermate no-treated mice as control group.

### Histological analysis

Freshly obtained skin samples from mice back with hair removal were fixed in 10% formalin or other fixatives for 12–24 hours at room temperature. Then were taken off water in 80%, 90% and 100% ethanol, two changes, 1 hour each. And add Xylene or xylene substitute for two times, 1.5 hours each. Put tissue into paraffin wax (58–60°C), two changes, 2 hours each. Embedding tissues into paraffin blocks. Place slides containing paraffin sections in a slide holder with xylene for 30 minutes to deparaffinize. Sections were rehydrated with 100% ethanol, 95% ethanol, 75% ethanol, deionized H_2_O, 3 minutes each. The Masson staining method can stain cells red and collagens within the dermal tissue in blue[16].Then use hematoxylin to stain nuclear and eosin to stain cytoplasm and visualized with a Leica microscope.

### Immunofluorescence analysis

Freshly obtained skin samples from mice back with hair removal were fixed in 4% paraformaldehyde for 8h. Then were taken off water in 10%, 20% and 30% sucrose gradient for 8h and embedded in Tissue Freezing Medium (SAKURA Tissue-Tek® OCT Compound). Frozen tissue sections of the skin were incubated with different primary antibodies at 4℃ overnight, which were anti-Akt (phosphoSer473, 1:200, GTX28932,GeneTex), anti- NF-κB (1:50, sc-8008,Santa Cruz), and anti-NF-κB (phoshoSer536, 1:100, 93H1, Cell Signaling Technology). Secondary antibodies with different fluorescence conjugates (FITC, TRITC) were used for the detection and visualized with an Olympus FV1000 confocal microscope.

### Western blotting

Freshly obtained skin samples from mice back with hair removal were prepared in a lysis buffer containing 1% Triton X-100, 1% deoxycholic acid, 2mM CaCl_2_ and protease inhibitors (10 μg/ml leupeptin, 10 μg/ml aprotinin, 1.8mg/ml iodoacetamide and 1mmol/l phenylmethyl sulfonyl fluoride) and quantified with a BCA protein assay kit (Pierce). Equal amounts of total protein were subjected to electrophoresis on 12% Bis-Tris gels, transblotted onto nitrocellulose membranes and probed with different primary antibodies: Anti-p53 (1:1000, sc-393031,Santa Cruz), anti-p16 (1:750, sc-1681,Santa Cruz), anti-p21 (1:250, sc-397,Santa Cruz),anti-GAPDH (1:1000, HC301-01, Transgen Biotech), anti-Akt (phosphoSer473, 1:5000, GTX28932,GeneTex), anti-Akt (Akt 1+2+3)(1:1500, GTX121937,GeneTex), anti-NF-κB (1:500, sc-8008,Santa Cruz), anti-NF-κB (phosphor Ser536,1:1000,93H1, Cell Signaling Technology), anti-Pten (phosphoThr366/pS370, 1:2000, GTX54620, GeneTex), anti-Pten (1:1000, 138G6,Cell Signaling Technology) respectively, followed by a peroxidase-conjugated secondary antibody (KPL). Immunoreactive bands were detected using ECL kit according to the manufacturer’s instructions. Subsequent reprobing using anti-GAPDH was performed for internal loading control.

### Real time PCR

Total RNA was extracted with TRIzol (TAKARA) following the manufacturer’s instructions. First-strand cDNA was prepared by PrimerScript^TM^ RT reagent Kit with g DNA Eraser (TAKARA) and oligo(dT) primers and stored at -20°C. Real-Time polymerase chain reaction (Real-Time PCR) was performed using SYBR® Premix Ex Taq™ II on an ABI 7300 QPCR System. As an internal control, levels of glyceraldehyde-3- phosphate dehydrogenase (GAPDH) were quantified in parallel with target genes. Normalization and fold changes were calculated using the ΔΔCt method. Primers used in the amplification of murine gene was as follows (Table 1):

**Table 1:** 

Genes	Forward	Reverse
*PTEN*	GGCTTCCGTCTGGAGGATTAT	AACCTGCCGAGATATTCCACA

### Statistical analyses

Results are expressed as mean ± s.e.m unless stated otherwise. Statistical comparisons between two groups were evaluated by Student's *t*-test, unpaired t-test, two-tailed. A probability (*P*) value of < 0.005 was considered to indicate statistical significance.

## Results

### The morphological and histological characteristics of the skin in mice of different age

**Fig 1:**
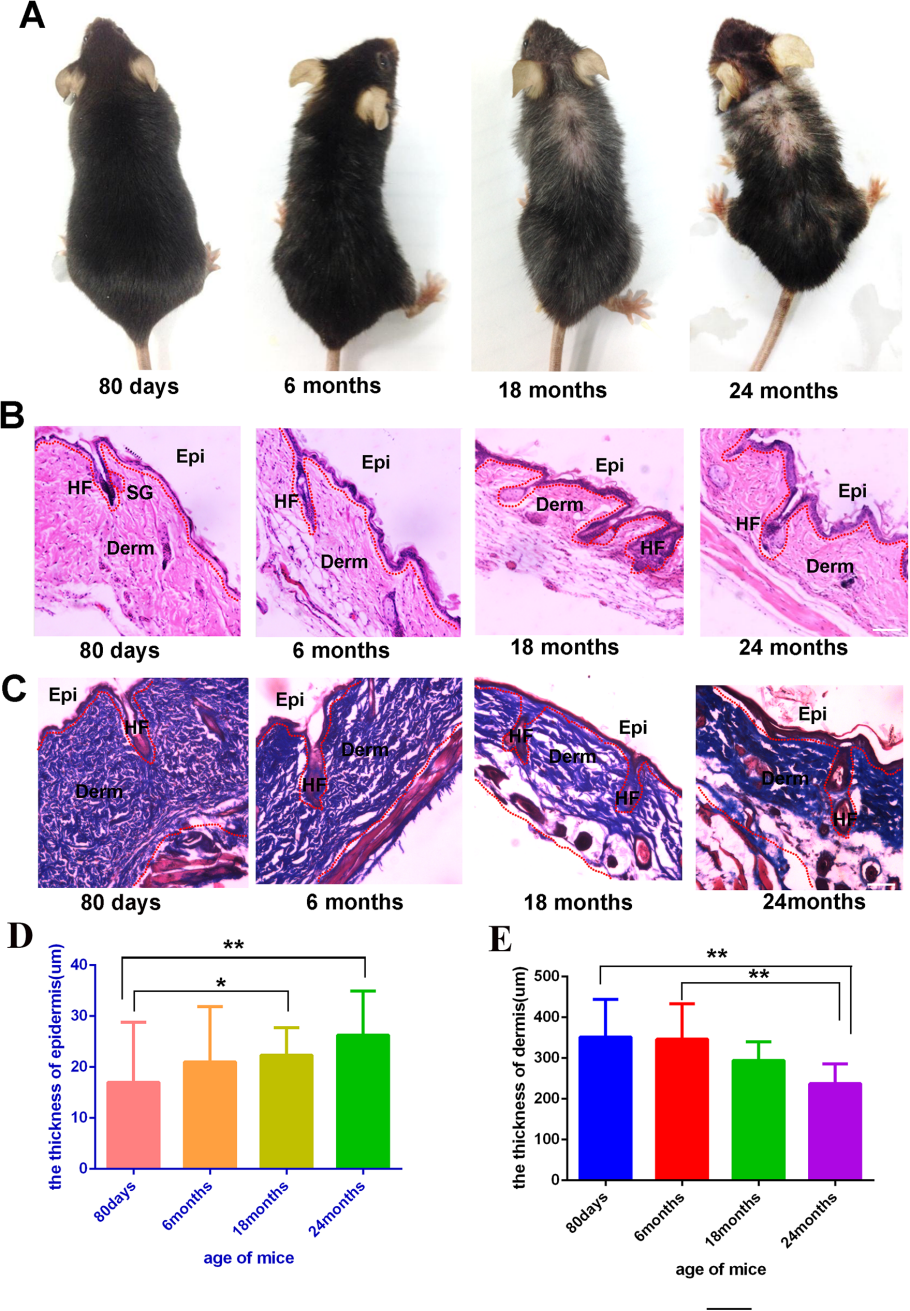
Representative images of mice of different ages, 80 days mice (n = 6), 6 months mice (n = 6), 18 months mice (n = 6), 24 months mice (n = 3) (A). HE staining of the skin from different ages mice (B,C), For HE and Masson staining, representative images from 8–16 tissue sections in 2–6 mice are shown the thickness of full skin and epidermis of different age mice(D,E).Data are expressed as the mean ± s.e.m. *** P<0.005, unpaired t-test, two-tailed. Scale bars, 50 μm.

Skin is a multifunctional organ, and alongside every other organ system, is subject to both intrinsic (chronological) and extrinsic (environmental) aging, resulting in a gradual loss of functional capacity, including the decline of functional capacity of the appendages in skin, such as hair follicles. Cutaneous aging can be observed from the appearance of the skin. For example, wrinkles and the appearance of white hairs can be seen as signs of cutaneous aging. In this study, the morphology of mice at different age was carefully documented. Firstly, as the major appendage organ of the skin, the hair follicle changed dramatically in both quantity and quality. As showed in Fig 1A, at age of 6 months (C57 mice), there is appearance of white hairs, and with increased aging the number of white hairs increased extensively. Furthermore, the quantity of hair decreased significantly after 18 months, and more so by month 24.

To further evaluate the histological changes during the progression of skin aging, histological examination of the skin in mice of different age was performed, the result shows that there was great reduction of skin thickness in mice aged 18 months and 24 months when compared to mice aged 80 days and 6 months. Further analysis found that the major cause of the thinner skin in elder mice was due to the reduction of adipocytes between the dermal layer and muscular layer. However, it is also obvious that the thickness of the epidermis is thicker in mice aged 18 months and 24 months than which mice aged 80 days and 6 months (Fig 1B). The Masson staining validate the thicker epidermis and thinner dermis of the aging mice skin (Fig 1C). Quantitative analysis of the thickness of the different tissue in skin was performed, and the results showed the epidermis of the mice aged 18 months and 24 months is much thicker than mice aged 6 months and 80 days. Where the mice aged 24 months is about two times thicker than the mice aged 80 days (Fig 1D). The thickness of the dermis of mice aged 80 days and 6 months is thicker than mice aged 18 months and 24 months (Fig 1E).Taken together, the data suggested that the dermal layer became thinner while the epidermal layer became thicker with aging.

### Both Akt and Pten activation levels were increased in the skin with aging

**Fig 2:**
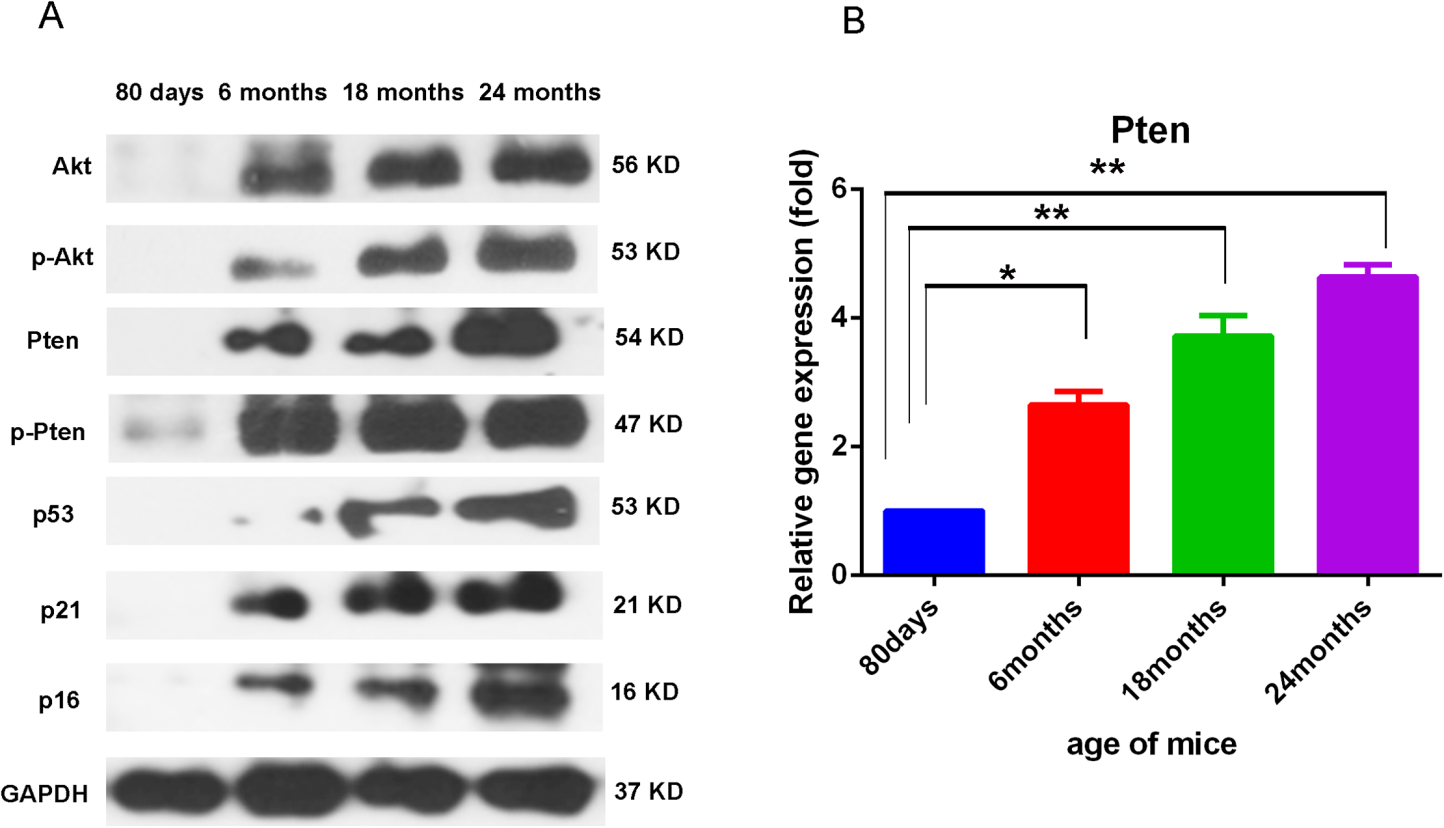
Akt activation levels in the skin of mice in different ages. Representative results of Western blotting analysis of skin tissues from mice aged 80 days, 6 months,18 months and 24 months for the levels of Pten, p-Pten, Akt, p-Akt (Ser 473), p53, p21 and p16(A). For all Western blot analysis, data are representative of 3–5 independent experiments. Real time PCR analysis of skins from different age mice showed higher PTEN expression with the mice age grows (B). For all real-time PCR analyses, gene expression was normalized to GAPDH with 40 cycles, data are represented as the mean ± SD, and N = 3.

To get further insight of the molecular feature of the skin in different mice at different age. Western blot analysis was performed, dorsal skin in full thickness of C57/B6 mice of 80 days, 6 months, 18 months and 24 months of age were collected and subjected to Western blot analysis, in which the level of Pten/p-Pten, Akt/p-Akt, p53, p21 and p16 were assayed and GAPDH was served as the internal control. Pten was the negative regulator of Akt activation, and the Akt activation was accomplished via the phosphorylation of Akt (p-Akt) at the aminol acid site of Thr308 or Ser473. p53 is a pro-apoptotic factor and is negatively regulated via Akt signaling[17]. p21 is a cell cycle inhibitory protein and could be inhibited by Akt via phosphorylate at Thr145 [18]. p16 exert antitumor potency and could downregulate the Akt signaling [19]. Western blotting analysis result showed that the expression of Akt, phospho-Akt (p-Akt, at Ser473) increased progressively in mice at 6 months, 18 months and 24 months of age. Unexpectedly, the expression level of Pten was also increased, suggesting that the enhanced Akt phosphorylation was not due to reduced inhibiting signals from Pten. Meanwhile, the expression of p53, p16 and p21 genes (involved with cell cycle regulation and aging) also increased markedly in a similar trend (Fig 2A). This data suggests that in the aged mice skin, a more complicated signaling regulation network exists. The crosstalk between Akt and Pten, p53, p16, p21 and other signaling pathways in aged mice skin still need further investigation.

Consistent with the results of the Western blotting, the Real time PCR analysis showed that the expression of PTEN increased with aging of mice skin, and its expression in the 24 months old mice is about 5 times higher than the 80 days mice. (Fig 2B).

### Immunofluorescence analysis of p-AKT in different aged skin

**Fig 3:**
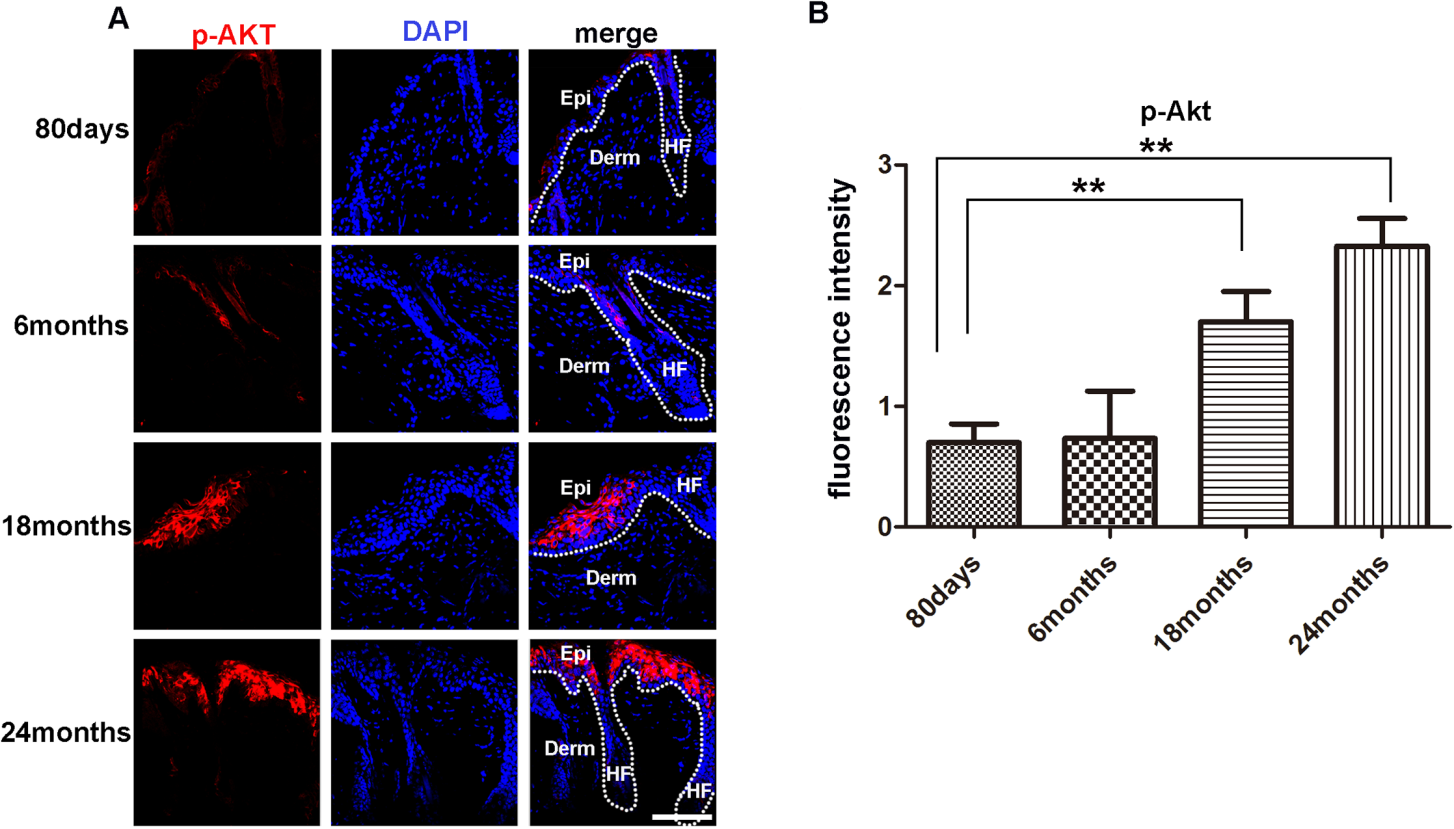
Levels of p-Akt in the skin of mice in different ages. (A)(IF?)Immuno-fluorescence analysis barely detected the presence of p-Akt positive cells in the skin of 80 days and 6 months old mice, but detected evident p-Akt positive cells in the epidermis and hair follicle of 18 and 24 months old mice. For all IF analyses, representative images from 8–16 tissue sections in 3–6 mice are shown. (B) Fluorescence intensity of p-Akt of 80days, 6months, 18 months, 24months old mice were measured by image J. Scale bars, 20 μm for microscopic images. Data are expressed as the mean ± s.e.m. *** P<0.005, unpaired t-test, two-tailed.

Certain signaling pathways that are exploited by tumor cells to promote cancer progression have been shown to play a fundamental physiological role in the regeneration of injured tissues [20]. At this point, the PI3K-Akt pathway is one of the most frequently anomalously activated intracellular signaling routes in cancer[21]. Studies have also shown that Akt activation promoted cutaneous wound repair via mTOR [22]. Suggesting a crucial role of Akt signaling in skin injury repair. Given the crucial role of Akt signaling in both cancer development and wound healing, it would be interesting to further explore the role of Akt signaling in aging skin. Here, immunostaining assay was firstly performed. Consistent with the increased p-Akt protein level in the Western blot analysis. The immunofluorescence staining analysis showed that p-Akt in the skin of 80 days and 6 months old mice was barely detectable, but abundant p-Akt positive cells was evidently found in the epidermis of 18 and 24 months old mice. Intriguingly, the p-Akt signal was predominantly detected in the epidermal layer. In contrast, p-Akt signal was barely detected in the dermal and hair follicles (Fig 3A). Quantitative analysis of the p-Akt signaling showed extensively increase of p-Akt signaling in 18 months and 24 months mice skin compared with the 80 day old mice skin (Fig 3B). This result indicates aging associated p-Akt signaling is mostly manifested in the epidermis, this is reminiscent for most tumors developed from the epithelium tissue, as the aging related p-Akt signaling in epidermal cells might be the promoting factor for tumor development.

### NF-κB signaling is activated in the aged mice skin

**Fig 4:**
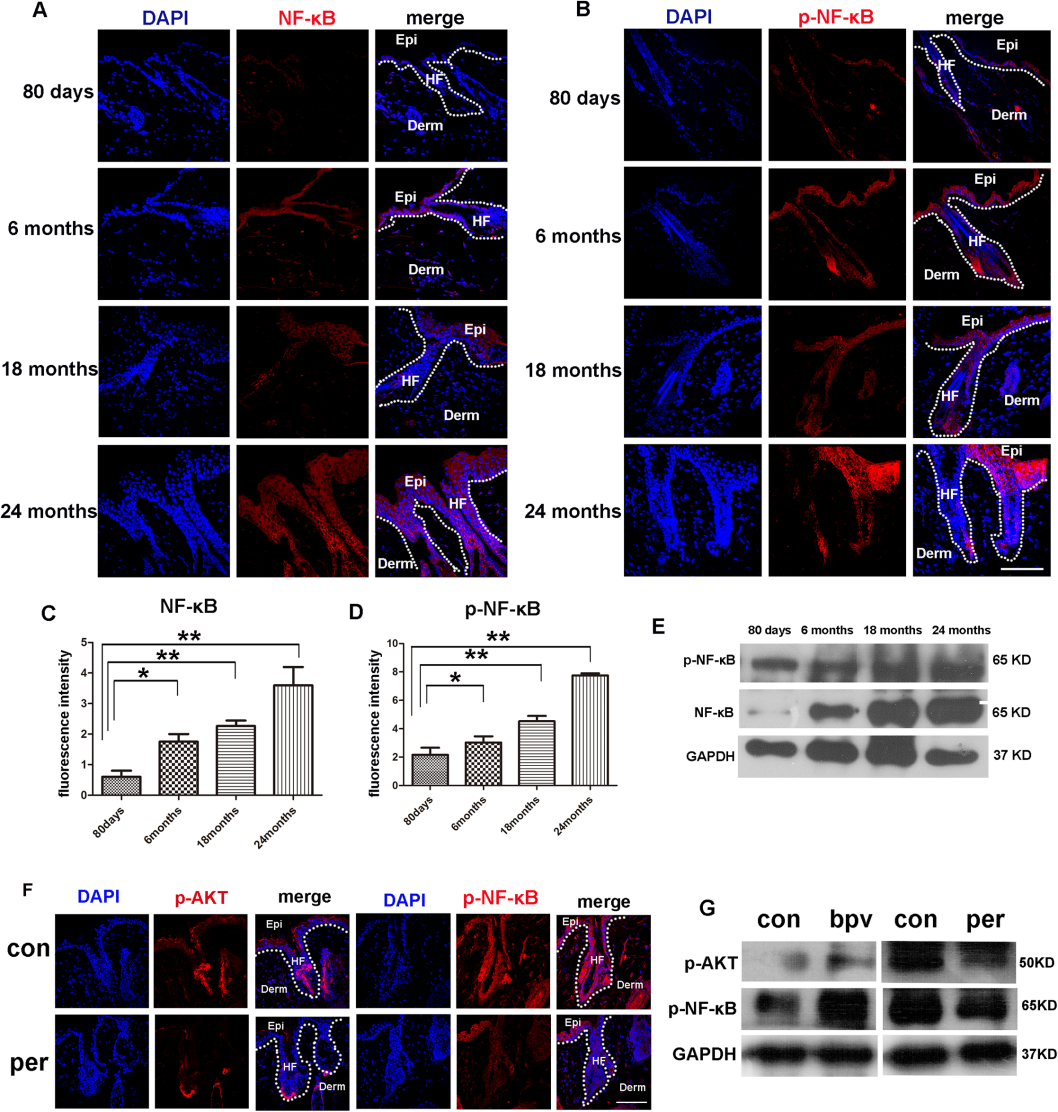
Levels of NF-κB, p-NF-κB in the skin of mice in different ages. (A, B) Immuno-fluorescence analysis showed increasing levels of NF-κB and p-NF-κB in the skin of mice with aging. Scale bars, 20 μm. (C,D) Fluorescence intensity of NF-κB, p-NF-κB of 80days,6months,18months, 24months old mice were measured by imageJ. (E) Western blotting analysis showed the levels of NF-κB and p-NF-κB in the skin tissue of mice aged 80 days, 6 months, 18 months and 24 months. (F) Immuno-fluorescence analysis showed increasing levels of p-AKT and p-NF-κB in the skin of 12 months mice treated with perifosine and the littermate without perifosine treated (N = 3). Scale bars, 20 μm. (G) Western blotting analysis showed the levels of p-AKT and p-NF-κB in the skin of 12 months mice treated with perifosine and 80 days mice which treated with the bpv(phen), the littermate no-treated mice as control group (N = 3).

The transcription factor NF-κB is broadly associated with oncogenesis because of its capacity to control cell proliferation and to suppress apoptosis. On the other hand, the NF-κB pathway in cancer has been associated with the control of metastasis and angiogenesis [23].The ser/thr kinase Akt can promote NF-κB activity, in which Akt functions through IKK to promote the transactivation potential and phosphorylation of NF-κB. In addition, Akt is proposed to promote tumor metastasis and angiogenesis through IKK, which depends on its downstream NF-κB and β-catenin activation[24]. Thus, NF-κB activity is closely related to the Akt signaling. To determine if the NF-κB activity is elevated in the aged mice skin, we examined the phosphorylated NF-κB in the skin via both immunofluorescence and Western blotting methods. The immunofluorescence result showed that in parallel with the increase of the p-Akt signaling, the levels of NF-κB and p-NF-κB increased progressively with aging. Interestingly, the age related NF-κB and p-NF-κB signaling activation could be obviously detected in both epidermis and hair follicles (Fig 4A and 4B). Quantitative analysis of the NF-κB and p-NF-κB signaling in different aged skin was shown in Fig 4C and 4D. The Western blot analysis analysis indicated that the level of NF-κB and p-NF-κB showed age related increase in a similar pattern as detected by immunofluorescence (Fig 4E). To further evaluate the regulation between AKT and NF-κB signaling, AKT signaling was inhibited via the Perifosine, a specific AKT inhibitor, both the immunostaining and western blotting assay showed that inhibition of AKT signaling result in the decrease of p-NF-κB signaling. In contrast, promotion of the AKT activity via bpv(Phen) result in elevated p-NF-κB signaling. These data indicating AKT regulate NF-κB in aging mice skin (Fig 4F and 4G). Collectively, in this present study, we systematically analyzed the histological feature of aging mice skin and detect the interesting activation feature of the Akt molecules. However, the crosstalk between these molecules still needs further exploration, our data do provide concrete molecular changes in the aged skin tissue and this will be valuable for further study of both molecular mechanism of aging and aging related tumor development.

## Discussion

Reduced level of Akt activity has been found in several organs and cells with aging, which is associated with decreased tissue regeneration ability [25, 26]. Contrary, to these observations, we showed that the levels of Akt expression and activation increased progressively in the skin with aging in mice, and the increased p-Akt level was largely co-localized to the epithelial cells in the epidermis and the hair follicle. The in vivo assay indicate the AKT signaling have a regulatory role in NF-κB signaling, while the crosstalk among AKT, NF-κB, p53, p16 and p21 is still illusive, further exploration the regulation of these signaling will come up with a clearer profile of the molecular mechanism of skin aging. To this end, a bioinformatics assay of the signal pathways at different aged mice skin will help us get a continuous molecular fingerprint in skin aging. Based on the molecular fingerprint, combined with the gain and loss function assay, we could further validate which signal has the skin aging promoting effect, and which have the aging inhibitory role.

Increased level of Akt activation in the aged skin is likely a consequence of elevated inflammation. It has been reported that with skin aging, the level of inflammation increase [27, 28]. Moreover, in this study, we observed that the level of Pten also increase in the aged skin, indicating that enhanced p-Akt was not due to a reduction of the inhibitory activity from Pten. The upregulation of Pten might be a response to the elevated level of Akt. Thus regulation between AKT signaling and Pten in skin aging still need further exploration. Given the gradually increased Pten expression in mice skin aging, and its capacity in AKT signaling inhibition, so we could suppose the Pten as the skin aging antagonist, which inhibit skin aging via down-regulating the AKT signaling in skin. Thus, it will be interesting to study the skin aging in the Pten knockout mice, which could specifically knockout Pten genes in the skin tissue.

The up-regulation of Akt activity may substantially change the behavior of the epithelial cells in the skin. Akt phosphorylates up to 100 substrates thereby modulating a variety of cellular activities including cell survival, proliferation and metabolism[27]. It has been reported that enhanced Akt phosphorylation in the skin epithelial cells cause epidermal hyperplasia and the development of cancer [6, 13]. Here in this study, we found increased levels of Akt activation in the aged skin, which may be a cause of increased incidence of skin cancer in the elderly.

In this study, we also found that the level of NF-κB activation increased in epithelial cells of the skin in aging mice. And the in vivo study suggest NF-κB activation is a consequence of increased Akt activation and inflammation, and there are other study describing the role of NF-κB in AKT signaling and inflammation. [29–31]. The immunostaining assay of study prove that the elevated AKT and NF-κB activities are mainly detected in the epithelial cells of skin. So what is the role of epithelial cell in skin aging, and could the epithelial aging driven the skin age, these are interesting question deserve further exploration. To this end, an in vitro assay more is desired, in which we could assay the epithelial cells aging in much efficient way, for example, a high glucose induce epithelial cells aging might be helpful.p53, p21 and p16 are tumor suppressor genes, which are critically involved in regulating cell growth, tumorigenesis and cell aging. In this study, we found that the expression levels of all these proteins increased in the skin with aging.
